# Efficient Transduction and Expansion of Ovine Macrophages for Gene Therapy Implementations

**DOI:** 10.3390/vetsci5020057

**Published:** 2018-06-18

**Authors:** Garyfalia Karponi, Spyridon Kritas, Evanthia Petridou, Eleni Papanikolaou

**Affiliations:** 1 Laboratory of Microbiology and Infectious Diseases, Faculty of Veterinary Medicine, Aristotle University of Thessaloniki, Thessaloniki 54124, Greece; aminoxy@yahoo.gr (G.K.); skritas@vet.auth.gr (S.K.); 2 Laboratory of Biology, School of Medicine, National and Kapodistrian University of Athens, Athens 11527, Greece

**Keywords:** zoonotic diseases, macrophages, ruminant animals, lentiviral vectors, transduction, gene therapy

## Abstract

A number of bacteria provoking zoonotic diseases present intracellular survival and a host cell tropism limited to the monocyte/macrophage lineage. Thus, infection is rendered difficult to eradicate, causing chronic inflammatory reactions to the host and widespread prevalence. Although self-inactivating lentiviral vectors have been successfully tested in the clinic against virally-induced human infectious diseases, little is known about the transduction susceptibility of ruminant animal phagocytes that play a critical role in the outbreak of zoonotic diseases such as brucellosis. In view of the development of a lentiviral vector-based platform targeting and inactivating specific genetic features of intracellular bacteria, we have tested the transducibility of ovine macrophages in terms of transgene expression and vector copy number (VCN). We show that ovine macrophages are relatively resistant to transduction even at a high multiplicity of infection with a conventional lentiviral vector expressing the green fluorescence protein and that addition of transduction enhancers, such as polybrene, increases transgene expression even after a one-week culture of the transduced cells in vitro. Overall, we demonstrate that ovine macrophages may be efficiently expanded and transduced in culture, thus providing the benchmark for gene therapy applications for zoonotic diseases.

## 1. Introduction

Bacterial infections in livestock not only pose severe public health issues, but also translate into a substantial financial burden for stockbreeders, due to reduction of the quality and quantity of the downstream products. Especially when the microorganism is able to survive intracellularly and evade the antibiotics’ effect, therapy efforts towards infected animals is cost ineffective and has an ambiguous benefit. Moreover, no effective vaccine currently exists for preventing such diseases in humans, while antibiotic therapy is long-lasting and often extends beyond six months. In addition, treatment in humans does not exclude relapse or re-infection after de novo exposure to the microorganism [1]. 

An illustrative example are bacteria of the genus *Brucella* that present a host cell tropism limited to the monocyte/macrophage lineage. Although immunized animals destroy *Brucella* via cellular immunity mechanisms [1], vaccination of herds, as well as their supervision is often ineffective, as the vaccine may lead to abortions and infertility in animals [2]. In addition, since the vaccine partially preserves its virulent properties, it is not entirely safe for clinical practitioners who administer it [2]. Moreover, the fact that the wildtype strain in seropositive animals is not distinct from the strain used in the vaccine, along with the long-term immune reaction that the vaccine may provoke in certain instances, renders the discrimination between vaccinated and infected animals impossible [3]. 

Consequently, the necessity of massive sanitation of ruminant livestock is crucial and indispensable. The scientific gap described above could possibly be overcome by gene therapy, a novel therapeutic strategy that utilizes non-pathogenic viral vectors to deliver normal genes into malfunctioning cells, in order to correct the pathological phenotype of genetic diseases. Gene therapy may be achieved with two different approaches [4]. In the first approach, the target stem cells are collected, co-cultured in the presence of a non-pathogenic viral vector to allow gene transfer to take place and are subsequently re-infused as a phenotypically functional graft back into the same patient from whom they were initially harvested. In the second case, gene correction takes place in the patient’s body, via systemic administration of the vector and is usually implemented in tissues in which stem cells cannot be readily characterized or isolated (e.g., lungs, brain, liver, etc.).

Over the last fifteen years, gene therapy has been successfully applied in clinical trials for monogenic diseases [5,6,7,8,9,10], providing a potentially lifelong therapeutic benefit to patients. Although gene therapy has also been successfully implemented at a clinical level in the field of infectious diseases, against hepatitis B virus (HBV), aiding in its clearance [11], and against human immunodeficiency virus-1 (HIV-1) [12], by inactivating the expression and replication of its genes, its effectiveness remains to be further evaluated. 

In order to facilitate massive sanitation of ruminant animal livestock by gene therapy, the development of a lentiviral vector-based platform targeting and inactivating specific genetic features of intracellular bacteria is considered essential. However, although the feasibility of gene transfer in human [13,14] or rodent animal [15] macrophages has been previously proven, little is known about the effectiveness of transduction in ruminant animal macrophages, for example of ovine origin. In the recent past, sheep have been effectively used as a model for plenty of gene transfer implementations targeting the nervous system [16,17], the respiratory tract [18], the eyes [19] or even for in utero gene therapy applications [20]. Moreover, lentiviral vectors have been extensively utilized to transduce small ruminant macrophages, but mostly in order to elucidate the immune responses against viral infections [21,22,23], rather than investigate the outcome of gene transfer itself. 

For the first time here, we analyzed the transgene expression and vector copy number (VCN) incorporated in the target cell genome, after exposure of peripheral blood-derived ovine macrophages to a conventional vesicular stomatitis virus glycoprotein (VSV-G)-pseudotyped lentiviral vector expressing the green fluorescence protein (GFP). We show that by the addition of transduction enhancers, such as polybrene or protamine sulfate, transgene expression may be further increased to high levels and that GFP expression may be sustained, under certain conditions, even after a one-week culture of the transduced cells in vitro. Overall, our findings demonstrate the feasibility of transduction in terms of cells of the monocyte/macrophage lineage of ovine origin and that it may prove useful for any gene therapy application against zoonotic diseases in the future.

## 2. Materials and Methods

### 2.1. Isolation of Ovine Monocytes

Approximately 25 mL of peripheral blood were collected from healthy sheep in controlled herds into ethylenediaminetetraacetic acid (EDTA)-anticoagulant vacutainers (BD, Franklin Lakes, NJ, USA) and tested for sterility after seeding in blood/agar dishes (Oxoid, Hampshire, UK). Complete blood counts (CBC) were also performed to ensure animals’ health status. The mononuclear cell fraction containing monocytes was separated through a Biocoll (Merck, Berlin, Germany) density gradient centrifugation at a ratio of 1 blood to 0.75 ficoll. Mononuclear cells were isolated, washed once with phosphate-buffered saline (PBS) (Lonza, Basel, Switzerland) and seeded in macrophage serum-free medium (Macrophage-SFM, Invitrogen, Carlsbad, CA, USA) supplemented with heat-inactivated fetal bovine serum (FBS) (Invitrogen, Carlsbad, CA, USA) as previously described [24]. Three weeks post culture, adherent cells were harvested and checked for CD14 expression by flow cytometry (BD FACSCalibur, Franklin Lakes, NJ, USA), after staining with the anti-bovine CD14 monoclonal antibody, conjugated with fluorescein isothiocyanate (FITC) (Bio-Rad, Hercules, CA, USA). An FITC-conjugated mouse IgG1 antibody was used as an isotype control (BD, Biosciences, San Jose, CA, USA). Acquisition data were analyzed using the CellQuest Pro v6 software (BD Biosciences, San Jose, CA, USA) or the Cyflogic software (www.cyflogic.com, CyFlo Ltd., Turku, Finland).

### 2.2. Macrophage Phagocytosis Assay

To determine the macrophages’ phagocytic activity, cells were exposed to green fluorescent polystyrene microspheres (Flow Cytometry Sub-micron Particle Size Reference Kit, Invitrogen, Carlsbad, CA, USA), each with a known diameter (0.2 μm, 0.5 μm, 1 μm and 2 μm), that show an excitation and emission profile similar to FITC and are thus collected by the FL-1 channel of the cytometer. The beads were added to approximately 10^5^ macrophages at a 1:1 ratio for 24 h. In order to quantify the phagocytosis events, the cells were washed 5–6 times with PBS, detached from the culture dishes with a cell scraper and analyzed by flow cytometry (BD FACSCalibur) using the CellQuest Pro v6 software (BD Biosciences, San Jose, CA, USA) or the Cyflogic software (www.cyflogic.com).

### 2.3. Lentiviral Vector Production and Titration

The vector used in this study was a self-inactivating (SIN) lentiviral vector that encodes GFP under the human phosphoglycerate kinase (PGK) promoter and also contains the 1.2-kb cHS4 insulator incorporated in the deleted region of U3. Details regarding the vector construct have been previously published [25]. Vector production and titration were performed as previously described [26]. Briefly, research-grade, VSV-G-pseudotyped lentiviral stocks of the GFP vector were generated by transfection of 293T cells with calcium phosphate precipitation using standard procedures. The final viral stocks were concentrated 100-fold by ultrafiltration. Serial dilutions of the concentrated viral stock were used to infect mouse erythroleukemia cells (MEL-585) for 3–4 days, and determination of the viral titer was documented by analyzing the cells for GFP expression by flow cytometry (BD FACSCalibur). Acquisition data were analyzed using the CellQuest Pro v6 software or the Cyflogic software (www.cyflogic.com). 

### 2.4. Macrophage Transduction

Ovine macrophages were seeded in 24-well tissue culture plates at a concentration of ~0.1 × 10^6^ cells/mL and were exposed, 24 h after initial seeding, to serial dilutions of the concentrated GFP vector stock corresponding to a multiplicity of infection (MOI) of 60, 6 and 0.6. To enhance transduction, 22 μg/mL hexadimethrine bromide (polybrene, Sigma-Adrich, St. Louis, MO, USA) or 37 μg/mL protamine sulfate (Sigma-Adrich, St. Louis, MO, USA) were added to the cells upon vector exposure. Two days later, cells were split and maintained in culture for another week. Transduction efficiency was assessed by monitoring the expression of GFP with flow cytometry, and vector-specific primers were utilized to determine the VCN per cell by quantitative real-time PCR. Mock-transduced cells and cells transduced in the absence of polybrene or protamine sulfate served as controls at all times. 

### 2.5. Vector Copy Number Analysis

Genomic DNA from cultured cells was extracted using the Nucleospin Tissue kit (Macherey-Nagel, Düren, Germany) as per the manufacturer’s directions. Quantification of the GFP lentiviral VCN was performed by real-time PCR based on SYBR green using vector-specific primers against the Rev response element (RRE) as previously described [26]. Average VCN per cell was calculated by normalizing to the endogenous ovine prion protein (Prp) single-copy chromosomal gene [27,28], in order to adjust for equal loading of genomic DNA per reaction, by using the following primers: ovPrpF 5’-GCCAAAAACCAACATGAAGCAT-3’ and ovPrpR 5’-TGCTCATGGCACTTCCCAG-3’.

### 2.6. Statistics

Multiple comparisons were performed using one-way ANOVA. Values of *p* < 0.05 were considered statistically significant. All results are expressed as the means ± the standard deviation (SD). 

## 3. Results

### 3.1. Characterization of Peripheral Blood-Derived Ovine Macrophages

**Figure 1:**
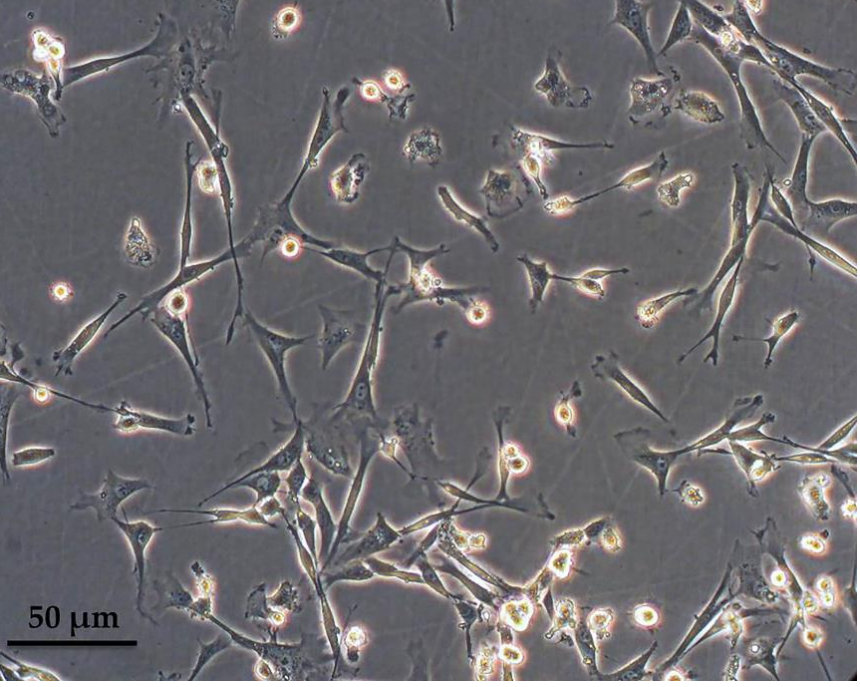
Ovine macrophages, after three weeks in culture. Cells were derived from the peripheral blood mononuclear fraction after ficoll separation and were left to mature in macrophage-specialized media supplemented with fetal bovine serum.

**Figure 2:**
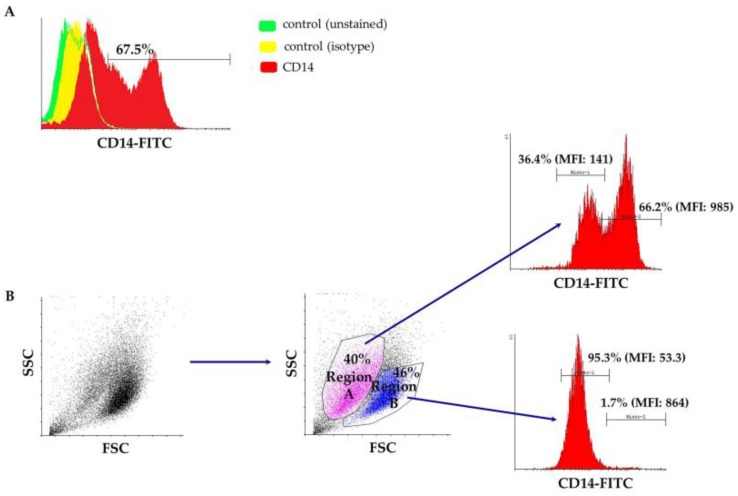
(**A**) A representative flow cytometry overlaid histogram showing the differentiation pattern of cultured cells towards the macrophage lineage (CD14^+^). Unstained cells (green histogram), as well as an FITC-conjugated mouse IgG1 isotypic antibody (yellow histogram) served as negative controls. (**B**) A representative flow cytometry dot plot depicting the two distinct cell populations that express different levels of the CD14 antigen. FITC: fluorescein isothiocyanate; FSC: forward scatter; SSC: side scatter.

Peripheral blood-derived mononuclear cells were isolated from the buffy coat after a ficoll density gradient centrifugation and cultured in macrophage-specialized media for three weeks, in order to allow for adequate maturation of ovine monocytes (Figure 1). Macrophages were phenotypically identified after staining with anti-CD14 and subsequent flow cytometry analysis and a mean of 67.5% of the population was found to express CD14 (ranging from 55–80%), as shown in Figure 2A from one representative experiment (n = 4). Interestingly, the culture was comprised of two, proportionally semi-equal cell populations with distinct size and granularity that also expressed the CD14 antigen to a different extent. More specifically, as indicated in Figure 2B, in the forward scatter/side scatter (FSC/SSC) dot plot, we consistently observed two well-defined populations: one population containing smaller and more complex cells, that we classified as Region A, comprising approximately 40% of the entire population, and a second population that was comprised of 46% of the cells, consisting of larger and less complex cells, classified as Region B. The vast majority of cells within Region B, i.e., 95.3%, expressed low levels of CD14 (CD14low) as evidenced by the mean fluorescent intensity of the CD14+ population that was on average 53.3 (Figure 2B). On the contrary, the larger-sized cohort, i.e., Region A, mostly was comprised of CD14^high^ cells (Figure 2B) since 66.2% of the cells were expressing high levels of CD14 (MFI: 985). To summarize, under the specified experimental conditions, we observed two different and distinct cell populations in terms of size and granularity. Larger and less complex cells (Region B) uniformly expressed low levels of CD14, whereas smaller and more complex cells (Region A) expressed both high levels of CD14 (66.2% of the cells in Region A, MFI: 985) and low levels of CD14 (36.4% of the cells in Region A, MFI: 141).

### 3.2. Phagocytosis Assay

**Figure 3:**
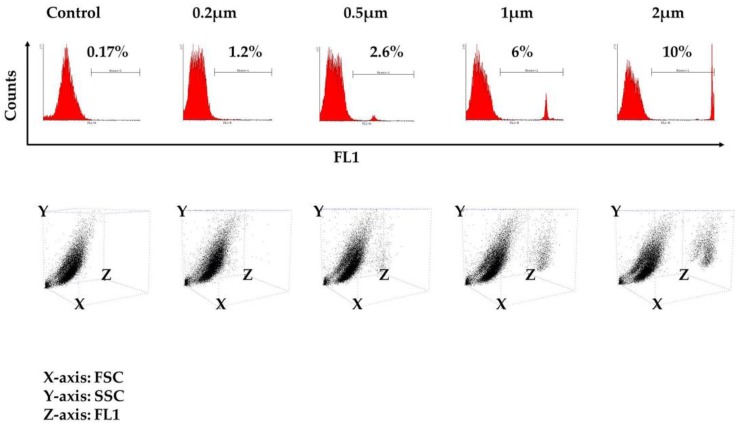
Macrophage phagocytosis assay. Results shown after flow cytometry analysis from one representative experiment. Histograms on the top illustrate the percentage of macrophages presenting phagocytosis events after a 24-hour exposure to variously-sized fluorescent beads (0.2 μm, 0.5 μm, 1 μm and 2 μm). The respective 3D dot plots are depicted on the bottom of the page. Note that both cell populations (i.e., from Region A and Region B) appear to possess phagocytic properties. Macrophages not exposed to beads served as a negative control. FSC: forward scatter; SSC: side scatter.

The kinetics of phagocytosis at the single-cell level was tested after exposure of the cells to variously-sized fluorescent beads ranging from 0.2 μm–2 μm in diameter that, upon engulfment, could be detected in the phagocytes’ cytoplasm with flow cytometry. The beads were added at a ratio of 1:1 (beads/cells), and as shown in Figure 3 from one representative experiment, 24 h after exposure to the beads, a certain percentage of the cells presented phagocytic properties, which proportionally increased with the diameter of the beads, as we noticed a higher percentage of phagocytosis when the 2-μm beads were utilized. A detailed description of the percentage of FITC-positive cells from each individual experiment is shown in Table 1 (control: 0.13% ± 0.04; 0.2 μm: 1.12% ± 0.11; 0.5 μm: 2.16% ± 0.45; 1 μm: 5.12% ± 1.32; 2 μm: 9.16% ± 0.74; number of experiments = 3). These findings confirmed that the cultured population is able to engulf beads of dimensions close to those found in bacteria, such as the 2-μm diameter, with greater efficiency. The 3D dot plot that is depicted in Figure 3 demonstrates that the ability to engulf is present in both populations of Region A and Region B.

### 3.3. Ovine Macrophages’ Resistance to Transduction May Be Averted with Transduction Enhancers

**Figure 4:**
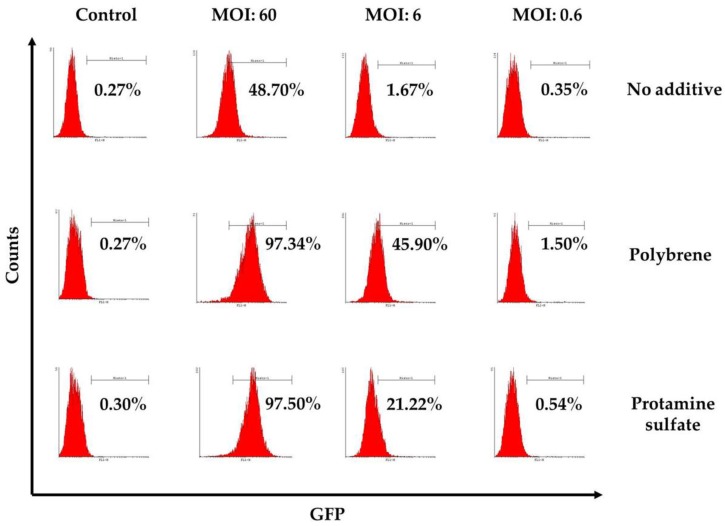
Representative flow cytometry results, three days post transduction with a conventional lentiviral vector expressing the green fluorescence protein in the presence or absence of transduction enhancers (polybrene or protamine sulfate). The percentage of GFP-positive cells is displayed on each histogram. Mock-transduced cells served as a negative control. GFP: green fluorescence protein.

**Figure 5:**
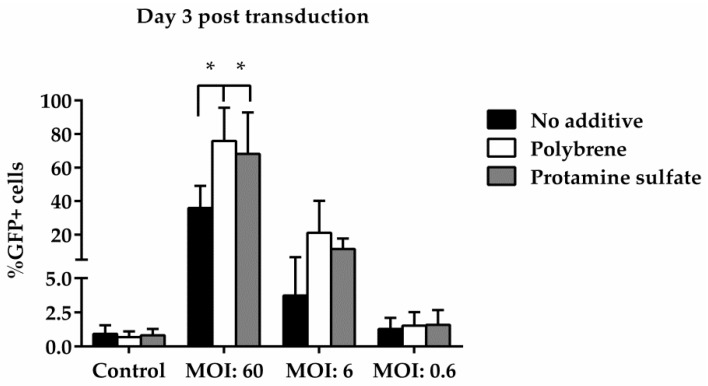
Comparative analysis of GFP expression in ovine macrophages, by flow cytometry, at various multiplicities of infection on Day 3 post transduction. Each MOI condition was repeated five times. Mock-transduced cells served as a negative control. GFP: green fluorescence protein; MOI: multiplicity of infection.

**Figure 6:**
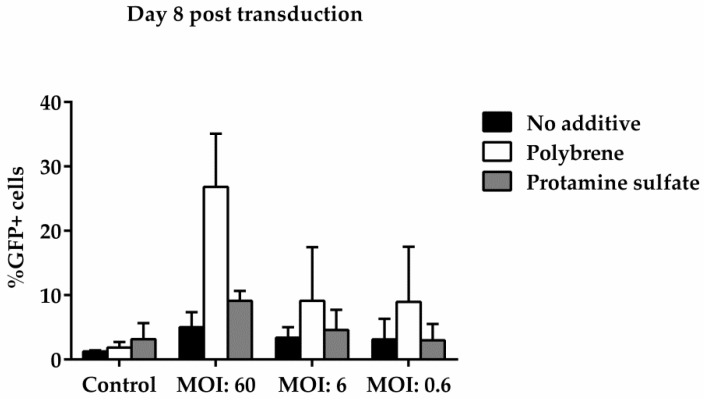
Comparative analysis of GFP expression in ovine macrophages, by flow cytometry, at various multiplicities of infection on Day 8 post transduction. Each MOI condition was repeated two times. Mock-transduced cells served as a negative control. GFP: green fluorescence protein; MOI: multiplicity of infection.

To examine ovine macrophages’ gene transfer efficiency, we exposed the cells to various concentrations of a GFP lentiviral vector and measured the expression of the green fluorescence protein, three and eight days post transduction. Our results demonstrate that even at the highest MOI of 60, ovine macrophages present a moderate transgene expression; a barrier, which, however, could be overcome by the addition of transduction enhancers, such as polybrene or protamine sulfate, at the time of vector exposure, as indicated in Figure 4 from one representative experiment (number of experiments = 5). A detailed analysis of the GFP-positive cells that were detected three days after transduction with the lentiviral vector is shown in Table 2 (no additive: control = 0.84% ± 0.65, MOI 60 = 38.6% ± 12.7, MOI 6 = 3.4% ± 3.03, MOI 0.6 = 1.12% ± 0.8; polybrene: control = 0.57% ± 0.38, MOI 60 = 79.9% ± 19, MOI 6 = 24.3% ± 19.3, MOI 0.6 = 1.14% ± 0.32; protamine sulfate: control = 0.74% ± 0.47, MOI 60 = 73.4% ± 23.5, MOI 6 = 12.6% ± 6.2, MOI 0.6 = 1.31% ± 0.99). Although both transduction enhancers showed a significant increase of gene transfer efficiency at the highest MOI on Day 3 (polybrene vs. no additive: 79.9% ± 19 vs. 38.6% ± 12.7, *p* = 0.01 and protamine sulfate vs. no additive: 73.4% ± 23.5 vs. 38.6% ± 12.7, *p* = 0.01) (Figure 5), only cells treated with polybrene displayed an increased GFP expression even at Day 8 post transduction, as compared with their no additive and protamine sulfate counterparts (Figure 6), demonstrating sustained transgene expression levels.

To complete the analysis, we calculated the vector copy number/cell (VCN) by real-time PCR. Our results showed that there was a dramatic decrease in VCN throughout the period of cultivation at the high MOI (MOI = 60). More specifically, although at Day 3, all experimental groups displayed high levels of gene marking, which was statistically significant particularly at the high MOI in comparison with the rest of the MOIs, we observed a severe decrease in VCN, at Day 8 post transduction (Table 3). With the exception of polybrene, which showed a VCN of two for MOI = 6 for Day 3 (data not shown), all of the MOIs and conditions failed to confer adequate gene marking eight days after transduction. This suggests that ovine macrophages, although they appear to be easily transducible by lentiviral vectors, fail to maintain a sustained VCN/cell after a certain period of time and are, thus, somewhat resistant to transduction. This feature precludes the possibility of gene addition via lentiviral vectors, but opens new perspectives for transient gene expression such as by employing genome editing tools, i.e., by transiently expressing components of the CRISPR/Cas9 system.

## 4. Discussion

Intracellular infectious agents such as bacteria of the genus *Brucella*, which affect ruminant animal populations, pose severe public health issues that are usually translated into a substantial financial burden for stockbreeders [29]. The only available means for eradicating the disease is based on vaccination programs, frequent monitoring and slaughtering of seropositive carriers [30]. In addition, the available animal vaccines are ambiguously effective, while, in addition, infectious for veterinarians [2]. Since application of programs for the eradication of brucellosis have been failing in southeastern Europe and the Mediterranean for almost four decades [31], massive sanitation of livestock is considered indispensable. Gene therapy, which has recently noted significant clinical successes in monogenic human disorders [5,6,7,8,9,10], as well as infectious diseases [11,12], could possibly constitute a novel, molecular-based platform for the development of a permanent cure not only for brucellosis, but for many other infectious diseases, where the microorganism presents an intracellular survival limited to the monocyte/macrophage lineage, thus effectively evading antibiotic treatment. For example, gene therapy could be utilized to render specific cell lineages resistant to infection, by deleting or inactivating genes that code for factors that play a critical role in the replication of the infectious agents in the host cytoplasm. This would be particularly feasible by introduction, via a lentiviral vector, of nuclease genetic information that would function specifically against the microbe genome, without harming the host DNA.

However, it is currently unknown whether macrophages of ruminant animal origin present a substantial susceptibility to transduction, so that gene therapy could be effectively implemented. Aiming at the development of a gene therapy approach targeting and inactivating specific genetic features of intracellular bacteria in our lab, we have tested the transducibility of ovine macrophages by lentiviral vectors in terms of transgene expression and VCN.

We first sought to determine whether the cells we isolated from the peripheral blood of healthy sheep could be cultured and expanded, so that an adequate number of macrophages could be generated for transduction. We show that the vast majority of cells in culture (~80%), in the presence of macrophage-specific media, did express the ruminant monocyte/macrophage CD14 differentiation antigen [32,33], to a various extent, which could be attributed to possible fluctuations in the differentiation status of the cells in culture. Extrinsic supplementation of growth factors, such as granulocyte-macrophage colony-stimulating factor (GM-CSF), to simulate in vivo maturation conditions [34] or the addition of interleukin 4/interleukin 13 to induce phagocytosis [35] may aid a more uniform CD14 expression. Further analysis of the surface antigens expressed or sorting of the populations may also elucidate the nature of these cells. Usually, phagocytosis is an effect that takes place within minutes [36] and is highly dependent on target shape and diameter [37]. Our results are in accordance with previously published literature that demonstrates that the maximal phagocytosis of polystyrene microspheres took place when their size was in the range of 1.0–3.0 μm. Thus, the absence of engulfment events in the cells exposed to the 0.2 μm-sized beads, along with the escalating percentage of phagocytic activity observed as the size of the beads increased, corroborates findings of other researchers [37].

It is widely acknowledged that macrophages, due to their germicidal nature, are particularly refractory to transduction [38,39]. Consequently, viral vector pseudotypes play a critical role in the transduction susceptibility of cells of the monocyte/macrophage lineage. In our study, we have utilized a classic VSV-G envelope, which allows infection to a wide range of cell types [40]. We report for the first time that sheep macrophages can be transduced with a conventional GFP lentiviral vector, but only under conditions of a high MOI and in the presence of cationic polymers, such as hexadimethrine bromide, which is commercially known as polybrene, or protamine sulfate. Both polybrene and protamine sulfate act by neutralizing the charge repulsion between virions and the cell surface [41,42]. Although they can be toxic to certain cell types, such as primary neurons [42], they are vastly used to increase the efficiency of transduction in cell cultures [43], if carefully titrated. In our case, we did not observe any toxic events associated with the use of polybrene or protamine sulfate, since, at all times, the viability of macrophages measured by trypan blue exclusion was over 90% (data not shown). This stands in accordance with a previous study from our group [26], where the same lentiviral vector, refined with an ultrafiltration concentration protocol as in our case, was used to transduce murine total bone marrow or human CD34^+^ hematopoietic stem cells, which resulted in equal transduction efficiency and reduced toxicity, compared to lentiviral vectors produced with standard ultracentrifugation-based methods.

Furthermore, polybrene-treated macrophages demonstrated a higher gene marking level even after culturing the cells for one week in vitro, although it should be noted that there was no statistical significance of the polybrene-treated cells against their no additive and protamine sulfate counterparts at the same time point, which most probably reflects the small number of samples analyzed. In addition, it is worth noting that the VCN per cell at the same time point was well correlated with the gene transfer rates measured by flow cytometry, since we observed higher GFP expression at the third day after transduction and a marked decrease both in VCN and GFP expression at Day 8 after transduction.

This finding is anticipated, in part, because lentiviral vector lots were derived from research-grade preparations, and the presence of transient plasmids contained in the vector stocks may lead to an overestimation of VCN at early time points post transduction. Thus, VCN is most often accurately measured when episomal particles are washed away upon subculturing of the cells. However, these results suggest that ovine macrophages although they appear to be easily transducible by lentiviral vectors, fail to maintain high VCN/cell after a sustained period of time and are, thus, somewhat resistant to transduction. This feature precludes the possibility of gene addition via lentiviral vectors, but opens new perspectives for transient gene expression such as by employing genome editing tools, i.e., by transiently expressing components of the CRISPR/Cas9 system in order to render the cells resistant to specific microorganisms.

In summary, the above data provide insights towards the generation of novel, target-specific molecular treatments against intracellular bacteria and are thus anticipated to provide the benchmark for gene therapy implementations of zoonoses.

## 5. Conclusions

We show that ovine macrophages are relatively resistant to transduction with lentiviral vectors even at a high multiplicity of infection and that addition of transduction enhancers, such as polybrene, increases transgene expression in vitro. Overall, we demonstrate that ovine macrophages may be efficiently expanded and transduced in culture, thus providing the benchmark for gene therapy applications for zoonotic diseases.

## Figures and Tables

**Table 1:** Percentage of FL-1 positive cells after 24 h of incubation with green fluorescent beads of different diameters.

	1st Experiment	2nd Experiment	3rd Experiment
Control	0.1	0.17	0.13
0.2 μm	1.15	1.21	1
0.5 μm	1.7	2.6	2.2
1 μm	3.6	6	5.75
2 μm	8.9	8.6	10

**Table 2:** Detailed results from all the experiments performed to determine the transduction efficiency. Numbers indicate the percentage of GFP-positive cells 3 days after transduction.

		1st Experiment	2nd Experiment	3rd Experiment	4th Experiment	5th Experiment
No additive	control	0.27	0.09	1.4	0.9	1.54
	MOI *:60	48.7	48.1	43.7	34.2	18.3
	MOI:6	1.67	1.66	8.8	2.7	2.37
	MOI:0.6	0.35	0.16	1.7	1.9	1.47
Polybrene	control	0.27	0.09	0.86	0.7	0.95
	MOI:60	97.34	97.46	78	75.6	51.4
	MOI:6	45.9	42.7	14.55	14.7	2.75
	MOI:0.6	1.5	0.71	1.34	1.25	0.91
Protamine sulfate	control	0.3	0.15	1.04	1.05	1.14
	MOI:60	97.5	94.7	70	64.7	40.3
	MOI:6	21.2	16.8	6.8	10.8	7.55
	MOI:0.6	0.54	0.41	1.06	2.8	1.76

* MOI: multiplicity of infection.

**Table 3:** Comparative vector copy number analysis of transduced macrophages, by quantitative PCR, at two different time points.

	Days Post Transduction	MOI: 60
No additive	3	10.46
	8	0.13
Polybrene	3	11.33
	8	0.29
Protamine sulfate	3	10.94
	8	0.21
